# Hypoglycemic Effect of Aqueous and Methanolic Extract of *Artemisia afra* on Alloxan Induced Diabetic Swiss Albino Mice

**DOI:** 10.1155/2015/752486

**Published:** 2015-08-05

**Authors:** Idris Ahmed Issa, Mohammed Hussen Bule

**Affiliations:** ^1^Department of Physiology, Faculty of Medicine, Mekelle University, Ethiopia; ^2^Department of Pharmacy, College of Medicine and Health Sciences, Ambo University, Ethiopia

## Abstract

Diabetes mellitus is metabolic syndrome that causes disability, early death, and many other complications. Currently insulin and many synthetic drugs are used in diabetes treatment. However, these pharmaceutical drugs are too expensive particularly for sub-Saharan population in addition to their undesirable side effects. The present study was aimed to evaluate antidiabetic effect and toxicity level of *Artemisia afra* which was collected from its natural habitat in Bale Zone, around Goba town, 455 km southeast of Addis Ababa. Air dried aerial parts of *Artemisia afra* were separately extracted with both distilled water and 95% methanol. Oral acute toxicity test was conducted on healthy Swiss albino mice. Antidiabetic effect of the aqueous and methanolic extracts of *Artemisia afra* was separately evaluated on alloxan induced diabetic mice at doses of 500, 750, and 1000 mg/Kg body weight orally. The results indicate that mean lethal dose (LD_50_) for aqueous extract of *Artemisia afra* was 9833.4 mg/Kg. Blood glucose level was significantly decreased by 24% (*p* < 0.005) and 56.9% (*p* < 0.0004) in groups that received aqueous extract of *Artemisia afra* at dose of 500 mg/Kg and 750 mg/Kg, respectively. The methanolic extract of *Artemisia afra* also significantly lowered blood glucose by 49.8% (*p* < 0.0001) at doses of 1000 mg/kg on the 5th hr. Aqueous extract of *Artemisia afra* was regarded as nontoxic and safe since its LD_50_ was found above 5000 mg/Kg. Aqueous extract showed higher effect at relatively lower dose as compared to methanolic extract. The aqueous extract was screened positive for phytochemicals like flavonoids, polyphenols, and tannins that were reported to have antioxidant activity.

## 1. Background

There is a global increase in the prevalence of diabetes mellitus predominantly, related to life styles and the resulting surge in obesity [1]. Diabetes mellitus is a metabolic disease characterized by disordered metabolism and abnormally high levels of blood glucose. It is a major public health problem affecting 285 million or 6.4% of the world population for the year 2010 [2]. Approximately, 90–95% of patients with diabetes have type 2 diabetes (T2D) or non-insulin dependent diabetes mellitus. T2D is accounting for a combination of insulin resistance and an inadequate compensatory insulin secretory response [3].

Management of diabetes without any side effect is still a challenge to the medical community. The use of the drugs is restricted by their pharmacokinetic properties, secondary failure rates, and accompanying side effects [4]. Despite considerable progress in the treatment of diabetes by oral hypoglycemic agents, search for newer drugs continues because the existing synthetic drugs have several limitations [5]. The available therapies for diabetes include insulin and oral antidiabetic agents such as sulfonylureas, biguanides, and *α*-glycosidase inhibitors. Many of these oral antidiabetic agents have a number of serious adverse effects. Thus, the management of diabetes without any side effects is still a challenge [6]. Therefore, there is a growing interest in herbal remedies because of their effectiveness, minimal side effects in clinical experience, and relatively low costs. Herbal drugs or their extracts are prescribed widely, even when their biological active compounds are unknown. Even the World Health Organization (WHO) approves the use of plant drugs for different diseases, including diabetes mellitus. Traditional plant medicines are used throughout the world for treatment of diabetes mellitus [7].

*Artemisia afra*, is locally called “chikugn” also known as African wormwood. It belongs to family Asteraceae. It is one of the best known and widely used plants in traditional medicine. Various preparations such as infusions, decoctions, molasses, and alcohol extracts of* Artemisia afra* are used for the treatment of coughs, colds, chills, stomachache, and dry dyspepsia and as a purgative and for the cure of smallpox and malaria. The herbaceous leaves of this plant contain between 0.3% and 1.4% v/w of a bluish-green essential oil, with a strong fragrance [8]. This study was designed to systematically evaluate hypoglycemic effect of aqueous and methanolic extracts of* Artemisia afra* in comparison with each other and standard hypoglycemic agents like glibenclamide.

## 2. Materials and Methods

### 2.1. Plant Material

Fresh aerial parts of* Artemisia afra* were collected during their flowering period when plants give highest yield [9]. The plants were collected from their natural habitat in Bale Zone, around Goba town, near the River Togona (7°0′N; 39°9′E), 2743 meters above sea level. Authentication and taxonomic identification of the plant samples were done by using standard botanical monographs at Addis Ababa University Science Faculty Herbarium.

### 2.2. Plant Extraction

#### 2.2.1. Aqueous Extraction

Fresh aerial parts of* Artemisia afra* were cleaned with distilled water and air dried at room temperature. It was ground into powder using grinding mill. Powdered aerial part of* Artemisia afra* weighing 220 gm was sorely mixed with 450 mL distilled water in flat bottom flask on orbital shaker for 24 hours. Distilled water was added until completely wet and the dried plant powder was sufficiently covered. After gentle maceration for 24 hours plant materials were filtered separately through mousseline cloth into 1000 mL beakers. The obtained filtrate was deep frozen at −70°C and lyophilized (freeze dried) using lyophilizer to collect crude aqueous extract [10]. The aqueous extracts were stored in desecrator at room temperature until used for experiment. The above aqueous extracted powders of* Artemisia afra* provided yield of 21 gm (9.5% w/w).

#### 2.2.2. Methanolic Extraction

Fresh aerial parts of* Artemisia afra* were cleaned with distilled water and air dried at room temperature. A dried and powdered aerial part of* Artemisia afra* weighing 165 gm was sorely mixed with 400 mL of 95% methanol in flat bottom flask on orbital shaker. After gentle maceration for 24 hours methanolic extracts were filtered through Whatman filter paper chart. The filtrate from* Artemisia afra* was concentrated under reduced pressure using rotary evaporator at 50 rpm and 40°C bath temperature. Finally concentrated methanolic extract was collected in vials and placed on water bath at 40°C to evaporate methanol completely. The crude methanolic extract of* Artemisia afra* had a yield of 13 gm (7.8% w/w).

### 2.3. Inducing Experimental Diabetes

Diabetic condition was induced to Swiss albino mice of both sexes, weighing 25–35 g after overnight fasting, by intraperitoneal injection of freshly prepared alloxan (200 mg/Kg, IP) dissolved in distilled water [11]. Three days (72 hr) after the injection of alloxan, the animals were fasted for 16 hours, and their blood glucose concentration was determined with glucometer. Animals showing stabilized diabetes (FBG ≥ 250 mg/dL) on the third day after alloxan injection were selected for the experiment [12]. Mice that did not show diabetic condition on the third day were excluded from the experiment.

### 2.4. Laboratory Animals

Healthy Swiss albino mice weighing 25 g–35 g were obtained from Ethiopian Health and Nutrition Research Institute (EHNRI) animal unit and kept in laboratory animal house of Drug Research Department during the experiment. The animals were provided standard pellet food and tap water* ad libitum* with 12-hour light/dark photoperiod. The experiment was conducted according to the ethical norms approved by Animal Ethics Committee Guidelines of the EHNRI.

### 2.5. Experimental Design

Alloxan induced experimental diabetic Swiss albino mice (FGB ≥ 250 mg/dL) were divided in to 9 test groups with 5 mice in each group that match with weight and fasting blood glucose level [13]. Groups-1–3 included diabetic mice treated with crude aqueous extract of* Artemisia afra* at dose of 500 mg/Kg, 750 mg/Kg, and 1000 mg/Kg PO. Groups-4–6 included diabetic mice treated with crude methanolic extract of* Artemisia afra* at dose of 500 mg/Kg, 750 mg/Kg, and 1000 mg/Kg PO. Group 7 included diabetic mice treated with standard drug glibenclamide (standard group) 10 mg/kg PO [14]. Group 8 included untreated diabetic mice (diabetic control) given equal volume of distilled water PO. Group 9 included nondiabetic reference group (normal control) given equal volume of distilled water PO. Blood glucose level of these mice was measured using glucometer at interval of 1 hour after treatment for consecutive 5 hr.

### 2.6. Acute Toxicity Test in Mice

The mice were divided into 10 groups with 6 animals in each group matched for weight and sex. These animals were housed 3 mice per sex per cage in a well-ventilated room with 12 hr cycle of day and night light conditions and temperature was maintained at around 25°C. Food was withdrawn for the 12 hr fasting period before and for 24 hr fasting period after oral administration of the single doses crude aqueous extract [12, 15]. Aqueous extract of* Artemisia afra* was aseptically suspended in distilled water and administered at a single doses of 0, 1000, 2000, 3000, 4000, 5000, 7500, 10000, and 12000 mg/Kg body weight by gavages perorally (PO) to the test groups. The control groups were given an equal volume of water. The change in general behaviors of the mice was continuously monitored [16]. The number of dead mice was recorded and used in the calculation of mean lethal dose (LD_50_) value. All surviving animals were euthanized with diethyl-ether at day 14 as described by [17].

### 2.7. Phytochemical Screening

Aqueous extract of* Artemisia afra* aerial parts was tested qualitatively for presence of major secondary metabolites. The qualitative test was done according to procedure described by [18]. The method was based on observation of color formation that occurs due to reaction of secondary metabolites with different standard reagents.

### 2.8. Statistical Analysis

All the results of blood glucose were expressed as mean ± standard deviation for (*n* = 5) animals in each group. The data was analyzed, using Microsoft Office Excel 2007, by Student's *t*-test at 95% CI.

## 3. Result and Discussion

### 3.1. Oral Acute Toxicity of* Artemisia afra*

As shown in the Table 1, the absence of mortality in the test group following oral administration of* Artemisia afra* aqueous extract up to 5000 mg/kg dose indicates the median lethal dose (LD_50_) of the* Artemisia afra* aqueous extract is higher than 5000 mg/kg. The expected median lethal dose (LD_50_) is between 7500 mg/Kg and 12000 mg/Kg. The actual median lethal dose (LD_50_) of* Artemisia afra* aqueous extract calculated from Table 1 was 9833.4 mg/Kg which agrees with the expected value. Since its actual median lethal dose (LD_50_) is greater than 5000 mg/Kg, aqueous extract of* Artemisia afra* is nontoxic. Substances with LD_50_ between 5000 mg/kg of body weight and 15000 mg/kg of body weight are regarded as practically nontoxic [19].

### 3.2. Hypoglycemic Effect of Aqueous and Methanolic Extracts of* Artemisia afra*

The aqueous extractof* Artemisia afra* was administered to alloxan induced diabetic mice orally at single doses of 500, 750, and 1000 mg/kg. Blood glucose level was significantly decreased in diabetic groups which received aqueous extract of* Artemisia afra* at dose of 500 mg/Kg and 750 mg/Kg in hours after treatment relative to untreated diabetic control with *p* < 0.01 and *p* < 0.001, respectively (Table 2). Among the tested doses of* Artemisia afra* aqueous extract, the maximal drop in blood glucose level of the diabetic mice was observed at dose of 750 mg/Kg (56.9%; *p* < 0.001). A sharp decline in blood glucose level at dose of 750 mg/Kg occurred in the first two hours after treatment.

In contrast the methanolic extract of* Artemisia afra* showed a remarkable blood glucose lowering effect in alloxan induced diabetic mice at doses of 750 and 1000 mg/kg as compared to the untreated diabetic control group (Table 2). Though the methanolic extract of* Artemisia afra* lowered blood glucose of diabetic mice by 25.7% and 49.8% at dose levels 750 and 1000 mg/kg, respectively, it brought statistically significant blood glucose lowering effect only at dose level of 1000 mg/kg (*p* < 0.001) as compared to the group receiving the vehicle.

**Figure 1:**
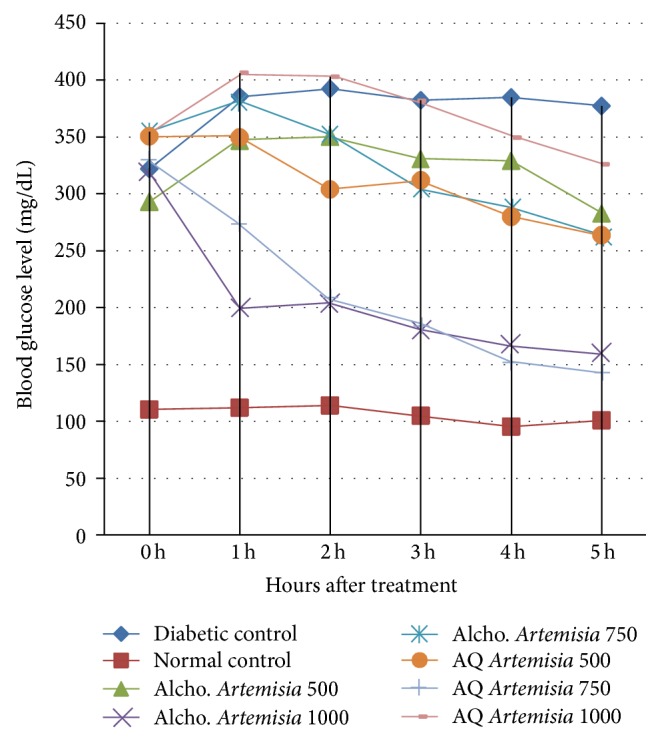
Comparison of hypoglycemic effect of aqueous and methanolic extracts of* Artemisia afra* at dose of 500 mg/Kg, 750 mg/Kg, and 1000 mg/Kg.

Unlike the aqueous extract of* Artemisia afra* which showed maximal effect at 750 mg/kg dose level the methanolic extract of* Artemisia afra* at this level did not significantly decrease blood glucose at this dose level (*p* > 0.05), Figure 1. Blood glucose level was initially increased in the 1st hr after treatment and started decreasing steadily from 2nd hr up to 5th hr of treatment in diabetic mice that received methanolic extract of* Artemisia afra* at 750 mg/kg dose level. Thus blood glucose level of diabetic mice did not come below 263.5 mg/dL throughout the 5 hr posttreatment period for this dose.

### 3.3. Phytochemical in Aqueous Extracts of* Artemisia afra* Aerial Part

The aqueous extract of* Artemisia afra* aerial part was screened positive for presence of chromophores, saponins, phosphosteroid and withanoids, flavonoids, tannins, and anthraquinone but showed negative result for presence of polyphenols, cyanogenic glycoside, anthranides, carotenoids, phenolic glycoside, cardiac glycoside, and alkaloids (Table 3).

## 4. Conclusion

The cute toxicity study indicates that the aqueous extract of* Artemisia afra* is found to be nontoxic through oral route, since the LD_50_ is greater than 5000 mg/Kg. Aqueous and methanolic extracts of this medicinal plant have significantly reduced blood glucose level in alloxan induced diabetic mice. Alloxan is known to induce free radical production and cause tissue injury, and the pancreas is especially susceptible to the action of alloxan induced free radical damage. It has been suggested that regeneration of islet beta cell following destruction by alloxan may be the primary mechanism of the recovery of alloxan-injected rats following drug administration [1]. Therefore, the extract could be inducing pancreatic cell regeneration. In addition, aqueous extracts of these plants were tested positive for presence of well-known antioxidant phytochemicals like flavonoids, polyphenols, and tannins. Therefore, this medicinal plant can be good candidate to be used as alternative treatment of diabetes. However intensive investigations must be conducted on the fractions of the extracts to identify pharmacological active compound(s), to elucidate mechanism of action, and to assure their safety and set appropriate dose. These herbs reduce blood glucose and may have beneficial effects on complications of diabetes.

As compared to methanolic extracts of* Artemisia afra*, the aqueous extracts of* Artemisia afra* showed a higher blood glucose lowering activity at relatively lower dose (750 mg/kg) than the methanolic extract at dose of 1000 mg/kg. This finding manifests the presence of the active ingredients in the aqueous extract at higher concentration than in methanolic extract, indicating the most active portions are polar.

## Figures and Tables

**Table 1:** Acute toxicity of *Artemisia afra *aqueous extract in Swiss albino mice (*n* = 6).

Dose of *A*. *afra* aqueous extract in mg/Kg	Number of mice per group (*n* = 6)		Number of mice dead and percent dead		Signs of toxicity
0.00 mg/Kg	Group-1	M = 3	0/3	0%	None
		F = 3	0/3		

1000 mg/Kg	Group-2	M = 3	0/3	0%	None
		F = 3	0/3		

2000 mg/Kg	Group-3	M = 3	0/3	0%	None
		F = 3	0/3		

3000 mg/Kg	Group-4	M = 3	0/3	0%	None
		F = 3	0/3		

4000 mg/Kg	Group-5	M = 3	0/3	0%	None
		F = 3	0/3		

5000 mg/Kg	Group-6	M = 3	0/3	0%	Hypoactivity, piloerection
		F = 3	0/3		

7500 mg/Kg	Group-7	M = 3	1/3	16.6%	Hypoactivity, piloerection
		F = 3	0/3		

10000 mg/Kg	Group-8	M = 3	1/3	33.3%	Hyperventilation, hypoactivity, and salivation
		F = 3	1/3		

12000 mg/Kg	Group-9	M = 3	3/3	100%	Convulsion, hyperventilation, hypoactivity, and salivation
		F = 3	3/3		

**Table 2:** Hypoglycemic effect of aqueous and methanolic extracts of *Artemisia afra* in alloxan induced diabetic mice (*n* = 5).

Treatment	Mean change of blood glucose (mg/dL) in hours after treatment (*n* = 5)						% reduction
	0 hr	1 hr	2 hr	3 hr	4 hr	5 hr	
Diabetic control	322 ± 6.3	385.5 ± 6.8	392.2 ± 5.9	382.25 ± 6.1	384.7 ± 6.2	377.2 ± 6.4	0.03%
AQ *Artemisia* 500	350.5 ± 3.4	349.7 ± 3.7	304 ± 3.2	311.75 ± 4.0	280 ± 3.8^*∗*^	263.75 ± 3.6^*∗*^	24%
AQ *Artemisia* 750	330.3 ± 2.2	274.3 ± 1.8	209.3 ± 1.6	186 ± 1.8^*∗*^	152 ± 1.5^*∗*^	142.3 ± 1.3^*∗*^	56.9%
AQ *Artemisia* 1000	351.7 ± 10.1	405.7 ± 9.8	402.7 ± 11.2	380.7 ± 10.6	349.5 ± 10.2	324.5 ± 9.9	7.6%

Meth. *Artemisia* 500	293.2 ± 7.7	346.7 ± 9.6	350 ± 5.2	330.5 ± 6.4	329 ± 6.1	283 ± 7.2	3.4%
Meth. *Artemisia* 750	354.25 ± 3.8	379.5 ± 4.0	351.7 ± 5.2	303.75 ± 4.6	287.75 ± 4.4	263.5 ± 3.5	25.7%
Meth. *Artemisia* 1000	319.5 ± 4.5	199.8 ± 5.0	204 ± 4.8	180.6 ± 2.2^*∗*^	166.5 ± 1.8^*∗*^	160.5 ± 2.2^*∗*^	49.8%
Normal control	110.5 ± 0.22	112 ± 0.21	114 ± 0.23	105 ± 0.28	95.25 ± 0.31	101 ± 0.25	8%

^*∗*^ *p* < 0.005 as compared to diabetic control.

**Table 3:** Results of preliminary phytochemical analysis in aqueous crude extracts of *Artemisia afra* aerial part.

Secondary metabolite	Reagents and method	Indicators	*Artemisia afra *
Chromophores	10 mL distilled water + heat for 30 min	Yellow to red color	+++
Polyphenols	1% FeCl_3_ + 1 mL of K_3_Fe (CN)_6_	Green blue color	− − −
Saponins	30 mL distilled water + heat (5 min)	Formation of honey comb froth	+++
Phytosteroids and withanoids	CHCl_3_ + conc. H_2_SO_4_	Red, reddish brown, or violet color	+++
Flavonoids	5 drops of 2% lead acetate	Yellow or orange color	+++
Tannins	3 drops of 1% K_3_[Fe(CN)_6_] + 3 drops of conc. NH_3_	Formation of color	+++

Anthraquinone glycosides	2 N HCL, benzene, 10% ammonia	Red color	+++
